# Development of a genetic tool for product regulation in the diverse British pig breed market

**DOI:** 10.1186/1471-2164-13-580

**Published:** 2012-11-15

**Authors:** Samantha Wilkinson, Alan L Archibald, Chris S Haley, Hendrik-Jan Megens, Richard PMA Crooijmans, Martien AM Groenen, Pamela Wiener, Rob Ogden

**Affiliations:** 1 The Roslin Institute and (Royal) Dick School of Veterinary Studies, University of Edinburgh, Easter Bush, Midlothian, EH25 9RG, UK; 2 MRC Human Genetics Unit, Western General Hospital, Crewe Road, Edinburgh, EH4 2XU, UK; 3 Animal Breeding and Genomics Centre, Wageningen University, Wageningen, The Netherlands; 4 Wildgenes Laboratory, Royal Zoological Society of Scotland, Edinburgh EH12 6TS, Scotland, UK; 5 Scotland's Rural College, The Roslin Institute Building, , Easter Bush, Midlothian, EH25 9RG, UK

## Abstract

**Background:**

The application of DNA markers for the identification of biological samples from both human and non-human species is widespread and includes use in food authentication. In the food industry the financial incentive to substituting the true name of a food product with a higher value alternative is driving food fraud. This applies to British pork products where products derived from traditional pig breeds are of premium value. The objective of this study was to develop a genetic assay for regulatory authentication of traditional pig breed-labelled products in the porcine food industry in the United Kingdom.

**Results:**

The dataset comprised of a comprehensive coverage of breed types present in Britain: 460 individuals from 7 traditional breeds, 5 commercial purebreds, 1 imported European breed and 1 imported Asian breed were genotyped using the PorcineSNP60 beadchip. Following breed-informative SNP selection, assignment power was calculated for increasing SNP panel size. A 96-plex assay created using the most informative SNPs revealed remarkably high genetic differentiation between the British pig breeds, with an average F_ST_ of 0.54 and Bayesian clustering analysis also indicated that they were distinct homogenous populations. The posterior probability of assignment of any individual of a presumed origin actually originating from that breed given an alternative breed origin was > 99.5% in 174 out of 182 contrasts, at a test value of log(LR) > 0. Validation of the 96-plex assay using independent test samples of known origin was successful; a subsequent survey of market samples revealed a high level of breed label conformity.

**Conclusion:**

The newly created 96-plex assay using selected markers from the PorcineSNP60 beadchip enables powerful assignment of samples to traditional breed origin and can effectively identify mislabelling, providing a highly effective tool for DNA analysis in food forensics.

## Background

The application of DNA analysis to the identification of biological samples has become routine in the fields of human [1] and non-human forensics [2], parentage analysis [3] and throughout the food industry. The ability to genetically authenticate the origin of food products is well established and has led to its use by industry to self-regulate, by eco-labels to promote sustainability and by government authorities to monitor the food supply chain and enforce legislation [4,5]. Mislabelling or substitution of food products can occur by accident or by intention, but it is widely recognised that knowingly substituting the biological name of a product with another (be it species, breed, variety and/or geographic origin) is widespread and is driven by strong financial incentives. This has been effectively illustrated in, for example, the fishing industry where widespread substitutions and mislabelling has been exposed using DNA evidence [2]. Agricultural production is also susceptible to food fraud, with examples from the United Kingdom (UK) ranging from the adulteration of basmati rice [6] and durum wheat pasta [7], to substitution within fruit juices [8]; all of which have been identified through the use of DNA techniques [4].

Within the UK, there has been a marked rise over the past decade in meat sold by breed, with traditional British livestock breed products attracting a premium price. This trend is exemplified by British pork products and there are several contributing factors to explain this consumer trend and the premium value of the product. Traditional pig breeds are slow growing, increasing production costs. The traditional breeds are also low in population size and the rarity makes them a more valuable commodity (Table 1). In addition, traditional British pig breeds possess certain meat qualities: high fat concentrations in the muscles and fine muscle grain [9]. These physiological attributes may contribute to an enhanced eating experience and increased preference for traditional pig breed meat. The enriched quality is not going unnoticed in the food industry; it is becoming common to see pork products labelled with a traditional pig breed names on restaurant menus, in supermarkets and at town farmers markets in Britain. For instance, Middle White pork is now a mainstay on the menus of top restaurants [10]. The increasing population sizes of the traditional pig breeds bears testimony to their rising popularity [11]. This trend has led to increased concerns over the authenticity of traditional breed meats, as the consumer is unlikely to be aware when substitution has taken place and fraud may therefore be perceived as a low risk crime. In addition to defrauding the consumer, breed mislabelling threatens the livelihoods of traditional breed farmers by undermining their brand and undercutting their prices through the illegal substitution with mass-produced meat.

**Table 1:** The British pig breeds

	**Breed**	**Sample size**	**Type**	**Status**^**1**^	**Sampling**^**4**^
1	Berkshire	73	Traditional	At Risk	PigBioDiv [15] and USA
2	British Saddleback	30	Traditional	Minority	PigBioDiv [15]
3	Duroc	31	Commercial		2 European and 2 USA populations [16]
4	Gloucestershire Old Spots	24	Traditional	Minority	PigBioDiv [15]
5	Hampshire	30	Commercial		PigBioDiv [15]
6	Landrace	30	Commercial		3 European and 2 USA populations [16]
7	Large Black	30	Traditional	Vulnerable	PigBioDiv [15]
8	Large White	34	Commercial		3 European and 2 USA populations [16]
9	Mangalica	26	European^2^		PigBioDiv [15]
10	Meishan	24	Asian^3^		PigBioDiv [15]
11	Middle White	30	Traditional	Vulnerable	PigBioDiv [15]
12	Pietrain	21	Commercial		2 European and 1 USA populations [16]
13	Tamworth	30	Traditional	At Risk	PigBioDiv [15]
14	Welsh	33	Traditional	At Risk	This study
		446			

^1^ population status quantified by the number of breeding females: Vulnerable = 300; At Risk = 500; Minority = 1000; taken from Rare Breeds Survival Trust ( http://www.rbst.org.uk/watch-list/pigs), ^2^ first imported to Britain from Hungary in 2006, ^3^ first imported to Britain from China in 1800s, ^4^ the sampling protocol is further described in the materials and methods.

There have been a number of genetic studies addressing the potential use of genetic markers for food authentication in livestock breeds through individual assignment analysis [12-14], which have been important in laying the groundwork for the use of DNA analysis to expose fraudulent food-labelling practices. However, these studies in essence, have been explorative and discursive: illustrating that DNA markers can be applied to food traceability, but without leading to the actual development of specific genetic assays. Whole genome sequencing and the availability of genome-wide Single Nucleotide Polymorphism (SNP) markers now permit the development of transferable and affordable genetic assays for DNA forensic analysis, particularly in non-human species [17]. The availability of dense genome-wide SNP markers provided in SNP chips for many livestock species offers the potential to develop genetic identification assays designed for regulatory purposes [18,19]. The PorcineSNP60 beadchip [16] can be exploited to authenticate British pig breed-labelled pork products and, in particular, samples allegedly originated from traditional pig breeds, being sold at a premium (Table 1).

With the aim of developing a genetic tool for the verification of meat from British traditional pig breeds for food authentication purposes, the objectives of this study were to: (1) select SNP markers that contain sufficient genetic information to be able to discriminate amongst the pig populations, (2) create a custom-made assay with an appropriate number of informative SNP markers, (3) demonstrate the effectiveness of the assay as a diagnostic tool, and (4) validate the application for product regulation.

## Results

### Selection of a breed informative SNP panel

**Figure 1:**
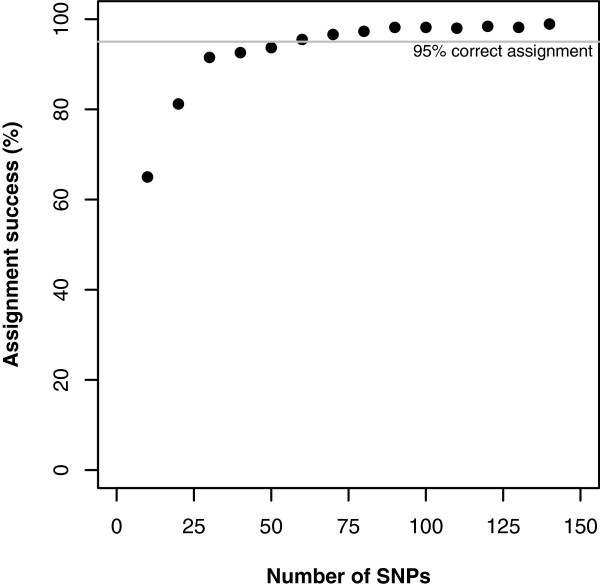
Plot of the individual assignment success for cumulatively increasing numbers of top-ranked informative SNP markers.

The power of the individual assignment test with cumulatively increasing number of top-ranked informative SNP markers is presented in Figure 1. With the top-ranked 50 SNP markers 93.7% of the individual genotypes (418) were correctly assigned. A 95% (426) assignment success was attained with 60 SNP markers. For 90 SNP markers the accuracy of individual assignment increased to 98.2% (438). The 8 incorrectly assigned individuals involved the following breed pairs: British Saddleback & Large Black (3), Landrace & Welsh (3), Landrace & Large White (1) and Middle White & Large White (1). For 140 SNPs, 98.9% of the individual genotypes (441) were correctly assigned (Figure 1). The 5 incorrectly assigned individuals involved Landrace & Welsh (4) and Middle White & Large White (1). Given the observed plateau of assignment success beyond 100 SNPs (Figure 1), the top 96 SNP markers were selected to form a marker panel for the subsequent production of a 96-plex genotyping assay. The names of the SNPs on the 96-panel are given, in decreasing order of informativeness, in Additional file 1: Table S1.

The genomic distribution of the final 96 SNPs is given in Table 2. As can be seen, the SNP markers were found on all chromosomes except for 2, 9, 10 and 18. The number of informative SNP markers selected from chromosomes ranged from 1 on chromosomes 12 and 17 to 25 on chromosome 8, with an average of 4 selected SNP markers per chromosome. The remaining 20 SNP markers have yet to be mapped to the porcine genome. A disproportionately large number of SNPs were located on chromosome 8 (Table 2). Paschou et al. [20] observed that panels of informative SNPs selected from genome-wide arrays tend to contain a large number of markers that are in high linkage disequlibrium (LD). This introduces redundant information into a panel because markers in complete LD will contain the same genetic information. The extent of LD between the 25 SNPs mapped to chromosome 8 was explored using Haploview [21]. Out of 600 marker pairs, 18 pairs exhibited moderate to high levels of LD in one or more pig breeds (*r*^2^ > 0.4; Additional file 1: Figure S1). The high levels of LD for each of the 18 marker pairs were not present in all 14 pig breeds, indicating that though a given marker pair may contain redundant information for one breed that is not necessarily the case for all breeds.

**Table 2:** Properties of the 96 SNP panel

**Chromosome**	**Occurrences**	**Distance (bp)**^**1**^
1	9	84,849,030 (97,690-221,777,480)
2	0	n.a.
3	2	10,181,626
4	4	20,026,279 (215,501-33,004,030)
5	5	4,864,199 (47,779-12,111,040)
6	2	18,925,439
7	6	8,294,008 (142,325-23,742,753)
8	25	14,218,857 (18,524-71,017,493)
9	0	n.a.
10	0	n.a.
11	4	15,622,017 (18,731-28,458,841)
12	1	n.a.
13	4	27,645,655 (39,053-55,278,293)
14	3	522,828 (127,875 – 784,243)
15	5	32,778,555 (91,640-81,029,219)
16	3	654,860 (70,990 – 982,290)
17	1	n.a.
18	0	n.a.
X	2	5,045,381
Undetermined	20	n.a.

^1^ The average distance between pairs of markers is provided with the minimum and maximum distance between pairs of markers in brackets.

### Assessment of the 96-SNP panel for genetic breed discrimination

**Figure 2:**
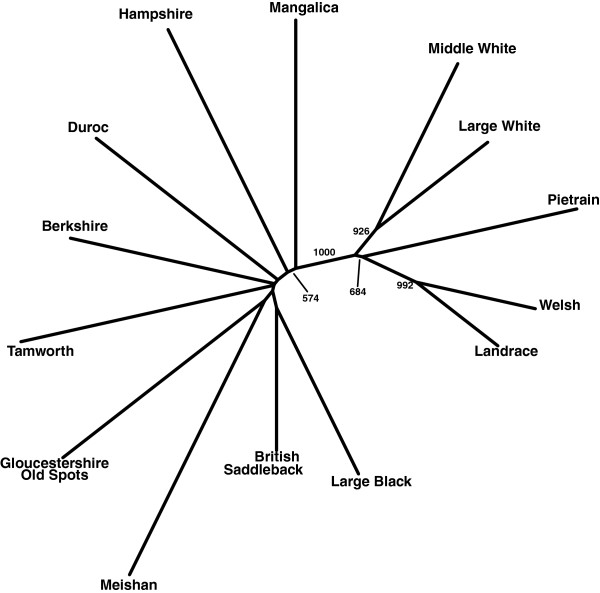
**Phylogenetic reconstructions of the British pig breeds using Reynold’s genetic distance.** Bootstrap support values greater than 50% are indicated.

Based on the reference data, the average pairwise breed genetic differentiation (F_ST_) using the 96-SNP panel was 0.54 (Table 3). The genetic differentiation (F_ST_) between pairs of breeds ranged from 0.10 (Landrace vs Welsh) to 0.82 (Hampshire vs Meishan), with average breed F_ST_ values ranging from 0.39 for British Saddleback to 0.71 for Meishan. Reynolds' pairwise genetic distance ranged from 0.34 between British Landrace and Welsh to 0.91 between Hampshire and Meishan. Average pairwise genetic distance across all breeds ranged from 0.63 for British Saddleback to 0.85 for Meishan. The phylogenetic reconstruction of breed relationships is shown in Figure 2 (bootstrap support > 50% indicated). There was high support for a clade of white-skinned breeds (Landrace, Large White, Middle White, Pietrain and Welsh) with additional support for some branching within the clade. For the remaining breeds, there was overall low bootstrap support for the depicted genetic relationships (Figure 2).

**Table 3:** Population genetic differentiation among 14 pig breeds using 96 SNP markers

	**Breed**	**BK**	**BS**	**DU**	**GLS**	**HA**	**LR**	**LB**	**LW**	**MA**	**MS**	**MW**	**PI**	**TA**	**W**	**F**_**ST**_
1	Berkshire															0.51 (0.10)
2	British Saddleback	0.29														0.39 (0.08)
3	Duroc	0.42	0.36													0.53 (0.08)
4	Gloucestershire Old Spots	0.47	0.43	0.56												0.62 (0.12)
5	Hampshire	0.51	0.45	0.60	0.75											0.64 (0.10)
6	Landrace	0.52	0.32	0.44	0.64	0.63										0.45 (0.19)
7	Large Black	0.39	0.23	0.50	0.43	0.56	0.53									0.50 (0.11)
8	Large White	0.56	0.35	0.50	0.67	0.60	0.19	0.55								0.46 (0.18)
9	Mangalica	0.51	0.40	0.57	0.68	0.63	0.53	0.52	0.50							0.58 (0.10)
10	Meishan	0.65	0.55	0.69	0.71	0.82	0.71	0.57	0.71	0.78						0.71 (0.08)
11	Middle White	0.64	0.45	0.60	0.75	0.69	0.36	0.61	0.22	0.63	0.78					0.56 (0.16)
12	Pietrain	0.63	0.46	0.59	0.77	0.71	0.30	0.64	0.33	0.63	0.81	0.47				0.57 (0.18)
13	Tamworth	0.45	0.43	0.53	0.61	0.66	0.62	0.45	0.64	0.64	0.75	0.70	0.74			0.61 (0.11)
14	Welsh	0.55	0.37	0.48	0.67	0.65	0.10	0.57	0.23	0.56	0.73	0.43	0.33	0.65		0.49 (0.19)

The lower diagonal contains the pairwise genetic differentiation between breeds estimated using Weir & Cockerham’s F_ST_[22]. The column on the far-right of the table presents the average breed F_ST_ and the standard deviation.

**Figure 3:**
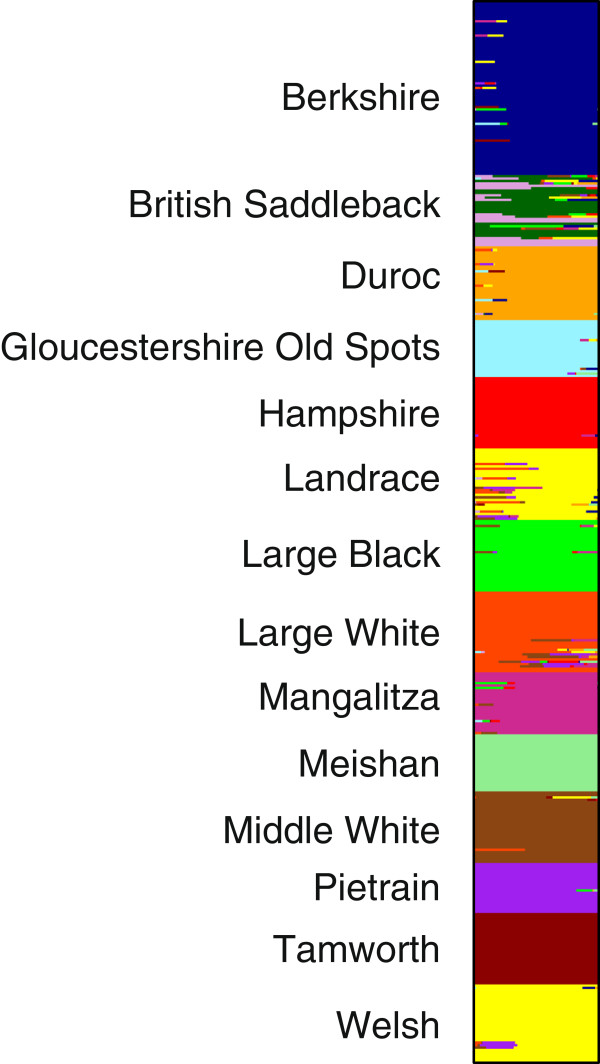
**Individual assignment based on BAPS analysis at K = 14.** The histogram demonstrates the proportion of each individual’s genome that originated from each of populations. Each individual is represented by a horizontal line corresponding to its membership coefficient (q).

The results of the BAPS analysis are presented in Figure 3. Given that there are 14 pig breeds sampled in this study, if all breeds were genetically distinct entities each pig breed would form an independent homogenous cluster for K = 14. However, at K = 14 the individuals of the Landrace and Welsh breeds clustered together, whilst the British Saddleback was split into two clusters. The other pig breeds were essentially distinct homogenous populations, with minimal evidence of genetic admixture (Figure 3). Large White and Middle White clustered together until K = 14, at which point they split to form separate clusters. The genetic subdivision in the British Saddleback breed was observed from K = 9. This breed substructure was also observed using microsatellite markers and was found to be associated with herds [23]. At K = 15, the Landrace and Welsh breeds still clustered together whilst Berkshire individuals split over two groups (mirroring the sampling of two geographic origins: USA and UK). Landrace and Welsh split at K = 16 to form two distinct clusters. A plot of the posterior likelihood against K values produced an asymptotic curve with a plateau that started at K = 15 and extended to K = 20 (at K > 16 the different populations within the commercial breeds split) (Additional file 1: Figure S2).

The exclusion-simulation test results are presented in Table 4. At a critical rejection region (α) of 0.001, 99.1% (442) individual genotypes could not be excluded from their reference population of origin. Those individuals excluded from their presumed origin (one each from Hampshire, Landrace, Large White and Pietrain breeds) were also excluded from all other reference populations.

**Table 4:** Exclusion-simulation analysis of reference populations

	**Breed**	**Number of samples excluded from breed origin**
1	Berkshire	0 / 73
2	British Saddleback	0 / 30
3	Duroc	0 / 31
4	Gloucestershire Old Spots	0 / 24
5	Hampshire	1 / 30
6	Landrace	1 / 30
7	Large Black	0 / 30
8	Large White	1 / 34
9	Mangalica	0 / 26
10	Meishan	0 / 24
11	Middle White	0 / 30
12	Pietrain	1 / 21
13	Tamworth	0 / 30
14	Welsh	0 / 33

### Expected power of the 96-SNP assay for pairwise breed discrimination

The posterior probability that any individual with a log likelihood ratio greater than a given threshold originated from the claimed breed origin rather than another specified breed, was calculated for all breed pairs at two thresholds (log(LR) > 0 and log(LR) > 2). At the test value of log(LR) > 0 the posterior probability of correct assignment was > 99.5% in 174 of the 182 and > 99.9% in 172 out of the 182 contrasts (Table 5). A posterior probability of correct assignment of below 99.5% of individuals to claimed breed was only observed in 4 breeds: Landrace, Large Black, Large White and Welsh. The remaining 10 breeds had a high level of assignment evident when contrasted against the other 13 breeds (Berkshire, British Saddleback, Duroc, Gloucestershire Old Spots, Hampshire, Mangalica, Meishan, Middle White, Pietrain and Tamworth). Three contrasts had a posterior probability of correct assignment below 99.0% at the test value of log(LR) > 0: Large White against Landrace (0.97), Landrace against Welsh (0.97) and Welsh against Landrace (0.91) (Table 5). At the test value of log(LR) > 2 the posterior probability of correct assignment was > 99.5% and > 99.9% in 175 of the 182 contrasts and 173 out of the 182 contrasts, respectively (Additional file 1: Table S2). There were 2 contrasts with a posterior probability of < 99.0% at test value of log(LR) > 2: Large White against Landrace (0.98) and Welsh against Landrace (0.95) (Additional file 1: Table S2). The lowest posterior probability of correct assignment at both log test values was the Welsh against Landrace contrast (Table 5, Additional file 1: Table S2).

**Table 5:** The posterior probability that any individual with log(LR) > 0 originates from the claimed breed

	**Contrasted breed**													
**Claimed breed**	**BK**	**BS**	**DU**	**GLOS**	**HA**	**LR**	**LB**	**LW**	**MA**	**MS**	**MW**	**PI**	**TA**	**W**
Berkshire	-	0.999946	1	1	1	1	1	1	1	1	1	1	1	1
British Saddleback	1	-	1	1	1	0.999997	0.999695	0.999612	0.999927	1	1	1	1	1
Duroc	1	0.999998	-	1	1	1	1	1	1	1	1	1	1	1
Gloucestershire Old Spot	1	1	0.999998	-	1	1	1	1	1	1	1	1	1	1
Hampshire	1	1	1	1	-	1	1	1	1	1	1	1	1	1
Landrace	1	0.991010	0.999570	1	1	-	1	0.998634	1	1	0.999989	0.999992	1	0.971693
Large Black	0.999968	0.990014	0.999968	1	1	1	-	1	0.999889	1	1	1	1	1
Large White	1	0.992750	0.999633	1	1	0.972057	1	-	1	1	0.994449	1	1	0.999673
Mangalica	1	0.999991	1	1	1	1	1	1	-	1	1	1	1	1
Meishan	1	1	1	1	1	1	1	1	1	-	1	1	1	1
Middle White	1	0.999747	1	1	1	0.999819	1	0.999401	1	1	-	1	1	1
Pietrain	1	0.999739	0.999967	1	1	0.999107	1	0.999805	1	1	1	-	1	0.999817
Tamworth	0.999996	1	0.999978	1	1	1	1	1	1	1	1	1	-	1
Welsh	0.999997	0.993296	0.998325	1	1	0.907609	1	0.999993	1	1	1	1	1	-

### Validation of the 96-plex assay using independent samples

#### *Control DNA*

The 96-plex Illumina Veracode™ assay allowed the unambiguous genotyping of 90 polymorphic SNP markers at each of the 70 test samples analysed; two SNPs failed to amplify and another four were monomorphic. In the double cross-validation analysis, 96% of the test samples were assigned to breed origin (Table 6). Only two breeds did not attain 100% assignment success, Landrace (2) and Middle White (1), for which test samples were assigned to Welsh and Large White, respectively. Identical assignment results were obtained for all control samples in the two laboratories.

**Table 6:** Exclusion-simulation analysis of independent test samples

		**Number of samples excluded from claimed breed origin**		
	**Breed**	**Test**	**Cooked**	**Market**
1	Berkshire	0 / 5	-	1 / 6
2	British Saddleback	0 / 5	-	0 / 3
3	Duroc	0 / 5	-	-
4	Gloucestershire Old Spot	0 / 5	0 / 6	2 / 10 (1 of which was assigned to another breed)
5	Hampshire	0 / 5	-	8 / 8 (1 of which was assigned to another breed)
6	Landrace	2 / 5 (to Welsh)	-	-
7	Large Black	0 / 5	-	0 / 1
8	Large White	1 / 5 (to Middle White)	-	-
9	Mangalica	0 / 5	^-^	^-^
10	Meishan	0 / 5	^-^	^-^
11	Middle White	0 / 5	0 / 6	0 / 3
12	Pietrain	0 / 5	-	-
13	Tamworth	0 / 5	0 / 6	0 / 4
14	Welsh	0 / 5	0 / 6	0 / 5

#### *Processed/treated meat samples*

Serial dilution of positive controls from 50 ng/μl down to 10 ng/μl showed that the performance of the assay was largely unaffected until the template DNA concentration reached 10 ng/μl, at which point genotyping rate and assignment accuracy fell off (data not shown). At 20 ng/μl there was no apparent loss of performance; to be conservative, the minimum DNA template concentration for this assay was set at 30 ng/μl. The performance of the assay following various cooking treatments (fried, baked, boiled, grilled, baked in sauce) showed correct assignment of all samples to their five breeds of origin, although the genotyping success rate (SNPs per sample) fell to a minimum of 88% (Table 6).

#### *Market samples sold by named breed*

Out of 40 market samples, the individual assignment analysis resulted in 2 samples not assigned to claimed breed origin but assigned to another breed, indicating possibly mislabelled meat (1 claimed Gloucestershire Old Spot and 1 claimed Hampshire sample; Table 6). While all 8 Hampshire samples were excluded from Hampshire reference population, 7 of the 8 samples were not assigned to any other breed. The assay failed to work on a number of sausage products, which was likely due to an insufficient yield of porcine DNA.

## Discussion

### Development of the 96-plex assay

The objective of this study was to develop a custom-made diagnostic genetic tool for the authentication of products originating from traditional British pig breeds and future regulation in the British porcine food industry. The availability of robust genotyping systems, where users can design their own multiplex assays using existing genetic markers, conveniently allows the achievement of this goal. In this study the GoldenGate Veracode™ system was used to develop the assay and certain pre-defined multiplex sizes were available: 48-, 96-, 144-, 192- and 384-plex. Careful analysis of the large number of markers available from the PorcineSNP60 beadchip indicated that the 96-marker assay would be sufficient to achieve a high level of assignment power. It was our assessment that more than 96 SNP markers did not sufficiently enhance the power of individual assignment analysis to warrant the development of a 144-plex assay for pork product authentication (Figure 1).

### The genetic power and utility of the 96-plex assay

It is important to establish whether the sampling of both genetic markers for the 96-plex assay and individuals for the British pig breeds were adequate, such that the developed assay and set of reference populations can be repeatedly used for future porcine food authentication. An earlier study using a panel of 50 microsatellites showed that European pig breeds are generally highly distinct populations [15]. One biological factor that could influence the levels of genetic differentiation amongst populations is hybridisation (cross-breeding). In the British pig breeds, very few individuals showed evidence of shared genetic ancestry, as revealed by Bayesian genotypic clustering analysis (Figure 3). The lack of evidence of genetic admixture within most populations and the genetic homogeneity of British pig breeds is consistent with previous work using microsatellite markers [23]. Strict breeding practices in Britain appear to maintain the genetic distinction of the pig breeds. This was further substantiated in this study where population genetic estimates demonstrated that the 96-plex assay was a highly effective selection of markers as it was able to genetically discriminate the British pig breeds. As can be seen in Figure 2, the predominantly long branches of breeds coupled with the high reported F_ST_ values are indicative of high breed genetic differentiation (Table 3). As a result of prior SNP selection, the 96-plex assay captured a large proportion of the genetic variation between the British pig breeds with estimates of F_ST_ exceeding those previously reported using a standard diversity panel of 50 microsatellite loci [15]. Although the high F_ST_ estimates of the SNPs on the 96-plex assay could be due to the process of random genetic drift, locus-specific breed genetic differences could also be a result of past artificial selection. A large proportion of the genetically informative SNPs were found on chromosome 8 (SSC8), which harbours the *KIT* gene, a locus involved in coat colour variation in domestic pig breeds. High linkage disequilibrium (LD) between some of these markers, especially in the commercial Large White and Pietrain breeds, could be a signature reflecting positive selection. This is in agreement with a recent genome wide study of commercial pig breeds in which low nucleotide diversity was found in regions of SSC8 [24]. High bootstrap support for the clustering of the white-skinned breeds using phylogenetic reconstruction in the current study was probably due to the selection of informative SNPs that are also associated with the *KIT* gene. Markers that show high breed differentiation due to positive selection for breed-specific characteristics may also be highly informative for breed assignment analyses.

The power of the individual assignment tests provided an indication that the breadth of actual genetic variation within each of the British pig breeds has also been effectively captured. That is, with sufficient numbers of individuals sampled, the estimated allele frequencies will provide a reasonable estimate of the actual population allele frequencies and, as a result, the individual assignment tests should perform well. The vast majority of the test samples used to validate the 96-plex assay were unambiguously authenticated, supporting the notion that the sampled breed populations are good representatives of the breeds (Table 6). The validation step was a vital exercise, not only to test the effectiveness of the SNP panel and the suitability of the reference population data, but also to demonstrate the application of the assay by a UK public analyst on case-type samples. It supported the accuracy and performance of the previous assignment tests and the overall low error rate indicates that the sampled British pig breed populations are genetically representative of the actual populations. The one possible exception to this was the observed lack of assignment in market samples of Hampshire. While it is not possible to determine if the failure was due to insufficient genetic diversity within the reference population or mislabelled test samples, in many countries the male Hampshire is often used to sire cross-bred pigs [10] and this practice could have altered the genetic composition of the breed to an extent that the reference Hampshire population (sampled in 1999) is not a good representative of the contemporary breed population. To investigate this issue, further reference samples of Hampshire pigs are being obtained for analysis and will subsequently be included in an additional validation study for this breed.

Although the prior selection of genetically informative markers allowed a high rate of correct assignment there were, nonetheless, a few instances of incorrect assignment of individuals. However, this was concentrated to a few breed pairings: the majority of the incorrectly assigned individuals were between the Landrace and Welsh breeds (Table 6). Relatively low genetic differentiation was observed between Landrace and Welsh with the 96-plex assay (Table 3, Figure 2). It would not be surprising to the pig breeding community that a close genetic relationship was observed between these two morphologically similar breeds. Dwindling numbers of the Welsh in the mid-20^th^ century resulted in the introduction of Landrace blood to boost the breed population size [25] and today the two breeds look remarkably similar. The results from this study show that the 96-plex assay does not allow differentiation of Welsh and Landrace pigs with sufficient accuracy for authenticity testing. Incorrect assignment also occurred in one case between Large White and Middle White (Table 6). Close genetic relationships between breeds need to be carefully considered in product authentication.

### The British pig breed market

The diversity of British pig breeds, expanding consumer preference and disparity in price between pork products create the potential for the substitution of labelled breed names in this food market. The conceivably profitable scenario of labelling a pork product with a traditional breed name when it actually originated from another source can be readily exploited. Therefore, it is in the interests of the food industry and consumer confidence to be able to verify traditional pig breed labelled products.

The 96-plex assay has the ability to authenticate pork products labelled with traditional breed names and thus expose mislabelled products. The levels of individual assignment accuracy were extremely high in the traditional breeds for both the reference populations and the test samples. More importantly, except for the Landrace/Welsh pairing, very few (commercial breed) individuals were falsely assigned to a traditional breed. Therefore, there is a high likelihood that an individual assignment test would assign a sample that was correctly labelled with a traditional pig breed name to that breed origin. Consequently, there was an extremely high probability of correct assignment for majority of the traditional pig breeds: Berkshire, British Saddleback, Gloucestershire Old Spots, Large Black, Middle White and Tamworth, particularly when contrasted against the other breeds (Table 5). Given the scenario that a food product labelled with one of these traditional pig breed names is in fact derived from another source then the probability of detecting such a swap is high.

Furthermore, the validation step of this study revealed a high level of breed label conformity across a range of samples tested for the traditional British pig breeds. The molecular technology of the 96-plex assay can be confidently applied to not only raw samples, but also meat subjected to various cooking treatments which is particularly relevant to verifying claims made on restaurant menus.

The power of the 96-plex assay as a genetic tool for British pig breed product authentication was only really compromised when confronted with Landrace and Welsh breed pair, as indicated by the notably reduced posterior probability of correct assignment (Table 5). A lower posterior probability of assignment of Welsh samples was obtained due to the relatively higher proportion of Landrace individuals falsely assigned to the former breed. These results are in concordance with the double cross-validation analysis in which two out of five Landrace individuals were assigned to the Welsh breed (Table 6).

This study illustrates the potential of the 96-plex assay to authenticate the origin of pork products labelled with traditional pig breed names. However, although commercial breed types were included in this study, in general commercially produced meat does not normally originate from purebred animals. Instead, commercial pork products are usually derived from lines that represent a broader cross of multiple from, perhaps including genetic components from traditional breeds. Although the 96-plex assay may be powerful at discriminating traditional pig breed from commercial pork products, actual samples from these crosses from a range of companies would need to be incorporated. This would then conclusively demonstrate that traditional pig breeds products may be discriminated form commercial pork products and validate the applicability of this genetic tool in the pork industry. Further sampling and analysis of commercial products is planned.

Another issue of cross-breeding is that some traditional breed products are sold as mixed ancestry. For instance, sometimes supermarkets explicitly label the breed of origin of the sire of meat, such that the named breed would attract a premium value to the product. In this study the assay was designed to authenticate pure-bred animals, rather than to identify the genetic make-up of cross-bred animals. While assignment results for falsely-labelled meat products of mixed ancestry and intentionally cross-bred animals may be predicted, in reality the assignment of unknown samples to multiple parental breeds is complex and beyond the scope of this test.

## Conclusion

The false labelling or mis-description of food is considered prevalent in the industry and the need to authenticate product origin is a long-standing challenge. The development of an Illumina Veracode™ 96-plex assay using markers available from the PorcineSNP60 beadchip will contribute to on-going product authentication and future regulation in the British food industry. This genetic tool provides a powerful method for authenticating products claimed to originate from traditional pig breeds.^a^

## Materials and methods

### Data

A total of 14 British pig breeds were used in this study (Table 1). The sample set comprehensively includes the two classification types of pig breeds (traditional and commercial) and majority of breeds of both types present in Britain [10]. Also included are the Meishan and Mangalica, two breeds of foreign origin that have been imported in high numbers to Britain (Table 1). By covering an almost complete spectrum of pig breeds present in Britain these dedicated samples have the potential to be used as custom sets for future food authentication investigations and regulatory purposes in the country’s porcine food industry.

A total of 446 individuals were genotyped using the PorcineSNP60 beadchip [16], which features ~60 000 SNPs with an estimated average density of one marker per 40 kb across the pig genome. Breed sample sizes ranged from 24 (in Gloucestershire Old Spots and Pietrain) to 73 (in Berkshire), with an average of 32 individuals genotyped per breed (Table 1). The majority of the breed DNA samples used in this study were previously extracted and genotyped using microsatellite loci as part of the PigBioDiv project, whereby breed sampling constituted a pair of siblings from 25 litters as unrelated as possible [15]. Additional samples in this study were collected from a separate Berkshire pig population in the U.S.A. and Welsh pigs.

Loci selected for analysis had a call rate of at least 80% across all the British pig breeds and in total 59,436 SNP from the 62,163 loci matched the call rate criterion. The individual multilocus genotypes were then used to identify genetically informative SNP markers and subsequently assess the genetic power of a selected panel of diagnostic markers chosen to create a custom-made genotyping multiplex assay.

### SNP selection and assay development

The genetic informativeness of each SNP from the PorcineSNP60 beadchip was estimated using delta, the allele frequency difference between a pair of populations [26]. The pairwise comparisons for each marker were averaged to obtain an overall estimate of the level of genetic information contained in each marker. It has been demonstrated that this approach can effectively identify markers that display high levels of dispersion in allele frequencies across a dataset when there are more than two populations being considered [19]. Such markers have relatively high levels of heterozygosity and have been shown to be highly efficient in population genetic assignment studies [27].

SNPs were subsequently ranked according to their delta value. To determine the numeric range of informative markers that would be appropriate for a custom-made GoldenGate Veracode™ multiplex assay, an individual assignment test was performed using cumulatively increasing numbers of top-ranked markers. A ‘self-assignment’ test, as described by Piry et al [28], was performed in GENECLASS2 using a partially Bayesian assignment method [29]. Prior to assignment testing of each individual, the observed allele frequencies of its respective reference population were re-estimated excluding the genotype in question, commonly referred to as the ‘leave-one-out’ validation method [30]. The likelihood of the multilocus individual genotypes occurring in each population was estimated based on their observed allele frequencies and an individual was assigned to a reference population for which it had the highest likelihood of assignment. If this was the known origin of the individual then the assignment test was deemed successful. This was a preliminary analysis to gauge the approximate number of markers that would be required and, consequently, the self-assignment test was used as it is straightforward to implement.

### Assessment of the assay for breed genetic discrimination

The performance of the selected informative SNP markers as the diagnostic marker panel for a custom-made 96-plex assay was assessed. The extent of population genetic divergence of the reference populations based on this assay was evaluated using a combination of traditional population genetic statistics and individual-based methods.

Weir and Cockerham’s unbiased estimator of Wright’s fixation index (F_ST_) [22] was calculated between pairs of breeds in FSTAT 2.9.3 [31]. Reynold’s genetic distance [32] was calculated between pairs of breeds using allele frequencies and consensus statistical support was calculated from 1000 bootstrap replicates using PHYLIP 3.67 [33]. An unrooted neighbour-joining cladogram was constructed from the genetic distance matrix of values for all pairs of breeds using the R package APE [34].

Population discrimination, group membership and levels of mixed ancestry in individuals were assessed using the Bayesian genotypic clustering method implemented in BAPS [35]. BAPS 5.2 uses a “greedy stochastic optimization” algorithm to first assign individuals to a population at a given K value and then to estimate the level of admixture in each individual (the membership probabilities for each individual being assigned to one or more clusters, measured by q) [35]. It operates by maximising Hardy-Weinberg Equilibrium and linkage equilibrium in the inferred clusters. Genetic clustering solutions were visualised in the statistical package R [36].

An exclusion-simulation test using a partially Bayesian method [29] was performed using GENECLASS2 [28]. For each reference population 10,000 independent individual genotypes were constructed from the observed allele frequencies. The likelihood that each simulated individual genotype was assigned to its respective reference population was calculated and a likelihood distribution for all 10, 000 simulated individuals for each reference population was constructed. The likelihoods of the individual genotypes were then compared to the distribution of likelihoods of simulated genotypes for each reference population. A critical rejection region (α) was implemented on the likelihood distribution such that an individual genotype was excluded from a population if the likelihood fell below the α * 10, 000^th^ lowest value of the distribution. Unlike the self-assignment test, under the exclusion-simulation method an individual genotype may be excluded from all reference populations; hence, it does not require that the population of origin is sampled.

### Power of the 96-plex assay for pairwise breed discrimination

The power of breed assignment using the 96-plex assay was also assessed by calculating the probability that an animal of an assigned breed was actually from that breed rather than from another breed. This allowed an assessment of probabilities of correct assignment in specific breed comparisons and was undertaken in order to represent a typical investigation in which there are specific claims and counter claims made concerning the breed origin of a pork product. The likely defence hypothesis that an observed individual genotype belongs to its designated breed origin (breed A) was tested against the likely prosecution hypothesis that an observed individual genotype in actuality belongs to another (breed B). If the defence hypothesis (that the observed individual genotype belongs to the labelled breed A) is rejected when it is in fact true, a Type I error has occurred (correct labelling undetected). If the defence hypothesis (that the observed individual genotype belongs to breed A) is accepted when it is in fact false, a Type II error has occurred (mislabelling undetected). Using these error rates, the posterior probability that a product is actually from breed A (its claimed breed origin) instead of from breed B can be estimated [13]. In brief, the log-likelihood that an individual originated from each breed was estimated in GENECLASS2 [28] as above and the log-likelihood ratio (log(LR)) of an individual originating from breed A versus breed B was calculated. The means and standard deviations of the observed log(LR) distributions were calculated and the false positives (*α*) and true positives (1 – *β*) were obtained for test values log(LR) > 0 and log(LR) > 2. Thus, the log(LR) of a positive result was estimated as the ratio between the likelihood of having a true positive result against the likelihood of having a false positive result: (1 – *β*)/ *α*, which gives the odds that the claimed breed origin (breed A) is correct when a test is positive. The posterior probability that an individual actually originated from breed A given the alternative hypothesis that it originated from breed B, assuming equal priors, was calculated as follows: (1 – *β*) / *α*) / ((1 – *β*) / *α*) + 1), which represents the proportion of individuals from claimed breed origin (breed A) correctly testing positive.

### Development and validation of the 96-plex assay using independent samples

Following selection of a panel of 96 SNP markers, a custom GoldenGate Veracode™ multiplex assay was designed and tested to assess its performance across a range of samples. Flanking regions from the original porcine SNP60 beadchip (which uses Illumina’s Infinium chemistry) were assessed using the Illumina Assay Design Tool for their suitability for conversion onto the Golden Gate chemistry. This assay was then produced and run against a set of control samples. (It should be noted that the original version of this assay suffered from relatively low SNP conversion success onto the Golden Gate format, resulting in an insufficient number of SNPs genotyping correctly and preventing subsequent breed identification. This led to a revised panel of 96 SNPs being selected and tested for breed differentiation *in silico*, before a second assay was produced for the validation study). In addition to the genotyping assay, a Standard Operating Procedure (SOP) was developed describing the downstream analytical process involved in assigning a sample to its most likely breed of origin.

Three sets of samples were used to validate and test the assay:

i. Control DNA from 70 samples from target breeds and comparative breeds at a concentration of 50 ng/μl (Table 1). These were included to demonstrate the ability of the assay to correctly assign samples to their breed origin. The work was replicated across two laboratories to ensure that the assay results and interpretation were reproducible by a second laboratory following the SOP.

ii. Processed/treated meat samples. These were included to examine the performance of the assay across a range of sample types, including various cooking methods (fried, baked, boiled, grilled, cooked in sauce) and serial DNA dilutions (50, 40, 30, 20, 10 ng/μl). Samples were obtained from the market sources (see above).

iii. Market/commercial samples sold by named breed. These were included as a final examination of how the assay would perform using market samples and to take an initial look at what breeds could be identified from a small sample of traditional breed products on sale in the UK. Samples of pork meat (pork chops unless otherwise stated) labelled by breed were purchased from 26 specialist suppliers and one supermarket by Minton Treharne & Davies Ltd, a Welsh Public Analyst involved in validating the assay. Names of individual suppliers are subject to confidentiality.

DNA from all samples was extracted using the Qiagen DNEasy Blood and Tissue kit following the manufacturer’s instructions and initially normalized to 50 ng/ul as suggested for the GoldenGate Veracode™ assay. DNA was then processed following the Illumina protocol and the data analysed using the proprietary GenomeStudio software. Following data QC, individual genotypes were exported for assignment analysis in GENECLASS2 (self-assignment and exclusion-simulation tests) and pairwise breed discrimination test, as described above.

## Endnote

^a^A Standard Operating Procedure (SOP) detailing the application of this method is available from the UK Department of Environment, Food and Rural Affairs (Foodauthenticity@defra.gsi.gov.uk).

## Competing interests

The authors declare no competing interests.

## Authors’ contributions

SW participated in the study design, wrote the computer code, carried out the statistical analysis and drafted the manuscript. ALA was a co-PI involved in project design. CSH was involved in manuscript preparation. HJM provided bioinformatics support and was involved in manuscript preparation. GMAM and RPMAC collected the samples and DNAs were extracted and genotyped in their laboratory. PW participated in the study design and manuscript preparation. RO was the Principal Investigator on the project, responsible for its conception, funding and implementation. All authors read and approved the final version of the manuscript.

## Supplementary Material

Additional file 1 **Table S1.** The top 96 informative markers present on the 96-plex assay listed in decreasing order of genetic informativeness. **Table S2.** The posterior probability any individual with log(LR) > 2 originates from the claimed breed. **Figure S1.** Level of linkage disequilibrium (LD), measured using *r*^2^, between the 25 markers on chromosome 8 for each pig breed. *r*^2^ represents the correlation of allele frequencies between two loci such that SNPs in complete LD have a value of 1. The darker the colour, the higher the LD with white indicating no LD between a pair of SNPs. **Figure S2.** Plot of the likelihood output of BAPS with increasing K value. Click here for file
