# Supplementary material for: Development of a genetic tool for product regulation in the diverse British pig breed market
Source: BMC Genomics. 2012 Nov 15;13:580. doi: 10.1186/1471-2164-13-580 (PMC3499217; doi:10.1186/1471-2164-13-580)
Supplement: Additional file 1 — Table S1. The top 96 informative markers present on the 96-plex assay listed in decreasing order of genetic informativeness. Table S2. The posterior probability any individual with log(LR) > 2 originates from the claimed breed. Figure S1. Level of linkage disequilibrium (LD), measured using r2, between the 25 markers on chromosome 8 for each pig breed. r2 represents the correlation of allele frequencies between two loci such that SNPs in complete LD have a value of 1. The darker the colour, the higher the LD with white indicating no LD between a pair of SNPs. Figure S2. Plot of the likelihood output of BAPS with increasing K value. [file 1471-2164-13-580-S1.doc]

Supplementary Table 1. The top 96 informative SNP markers present on the 96-plex assay listed in decreasing order of genetic informativeness.

|  | Marker name | Index number | Chromsome | Position |
| --- | --- | --- | --- | --- |
| 1 | ALGA0106261 | 15864 | Unknown | 0 |
| 2 | ALGA0108039 | 16309 | Unknown | 0 |
| 3 | ALGA0108841 | 16511 | Unknown | 0 |
| 4 | ALGA0117575 | 18582 | Unknown | 0 |
| 5 | ASGA0104113 | 34887 | Unknown | 0 |
| 6 | DIAS0000042 | 36444 | Unknown | 0 |
| 7 | DIAS0000043 | 36445 | Unknown | 0 |
| 8 | DIAS0000661 | 36666 | Unknown | 0 |
| 9 | H3GA0053839 | 46769 | Unknown | 0 |
| 10 | H3GA0056129 | 47189 | Unknown | 0 |
| 11 | M1GA0026964 | 51703 | Unknown | 0 |
| 12 | MARC0024767 | 54814 | Unknown | 0 |
| 13 | MARC0029724 | 55450 | Unknown | 0 |
| 14 | MARC0030810 | 55597 | Unknown | 0 |
| 15 | MARC0046321 | 57479 | Unknown | 0 |
| 16 | MARC0051265 | 57968 | Unknown | 0 |
| 17 | MARC0061507 | 59164 | Unknown | 0 |
| 18 | MARC0084160 | 61274 | Unknown | 0 |
| 19 | MARC0084409 | 61288 | Unknown | 0 |
| 20 | MARC0101493 | 62904 | Unknown | 0 |
| 21 | ALGA0001762 | 210 | 1 | 19689099 |
| 22 | ALGA0003067 | 411 | 1 | 38370130 |
| 23 | ALGA0003076 | 414 | 1 | 38467820 |
| 24 | ALGA0003145 | 431 | 1 | 40280404 |
| 25 | ALGA0010391 | 1942 | 1 | 236196501 |
| 26 | ALGA0010777 | 1980 | 1 | 241466579 |
| 27 | INRA0002279 | 47421 | 1 | 43287493 |
| 28 | INRA0002835 | 47456 | 1 | 60087556 |
| 29 | MARC0025593 | 54900 | 1 | 43734434 |
| 30 | ALGA0018563 | 3018 | 3 | 12799523 |
| 31 | ASGA0014878 | 22147 | 3 | 22981149 |
| 32 | ALGA0026051 | 4182 | 4 | 74388002 |
| 33 | ASGA0019578 | 22730 | 4 | 41599473 |
| 34 | H3GA0013097 | 42500 | 4 | 74603503 |
| 35 | INRA0014142 | 48163 | 4 | 53242418 |
| 36 | ALGA0031516 | 4909 | 5 | 24224087 |
| 37 | ALGA0031527 | 4915 | 5 | 24882661 |
| 38 | ASGA0024792 | 23340 | 5 | 12771621 |
| 39 | ASGA0025238 | 23411 | 5 | 24176308 |
| 40 | DRGA0005608 | 38776 | 5 | 24275222 |
| 41 | ALGA0036101 | 5581 | 6 | 18204577 |
| 42 | INRA0022517 | 48476 | 6 | 37130016 |
| 43 | ALGA0042019 | 6343 | 7 | 56538924 |
| 44 | ALGA0042134 | 6355 | 7 | 58219628 |
| 45 | ALGA0042589 | 6485 | 7 | 79901878 |
| 46 | DRGA0007655 | 39166 | 7 | 56681249 |
| 47 | H3GA0021745 | 43324 | 7 | 56159125 |
| 48 | MARC0059240 | 58950 | 7 | 57335493 |
| 49 | ALGA0047109 | 7118 | 8 | 12263285 |
| 50 | ALGA0047798 | 7208 | 8 | 19593136 |
| 51 | ALGA0047813 | 7213 | 8 | 20029206 |
| 52 | ALGA0047848 | 7224 | 8 | 22487301 |
| 53 | ALGA0047859 | 7227 | 8 | 22846526 |
| 54 | ALGA0047912 | 7246 | 8 | 26164140 |
| 55 | ALGA0048114 | 7310 | 8 | 34700891 |
| 56 | ALGA0048142 | 7318 | 8 | 35372064 |
| 57 | ASGA0038785 | 24997 | 8 | 20153485 |
| 58 | ASGA0038901 | 25022 | 8 | 33360642 |
| 59 | INRA0029810 | 48740 | 8 | 21967480 |
| 60 | INRA0029890 | 48754 | 8 | 33379166 |
| 61 | INRA0029891 | 48755 | 8 | 33492037 |
| 62 | INRA0029897 | 48756 | 8 | 34000158 |
| 63 | MARC0003787 | 52206 | 8 | 83280778 |
| 64 | MARC0006407 | 52561 | 8 | 22969532 |
| 65 | MARC0007151 | 52663 | 8 | 22792312 |
| 66 | MARC0024662 | 54806 | 8 | 24665960 |
| 67 | MARC0038980 | 56638 | 8 | 23259080 |
| 68 | MARC0053405 | 58235 | 8 | 19787030 |
| 69 | MARC0056888 | 58672 | 8 | 23810304 |
| 70 | MARC0071439 | 60125 | 8 | 24464495 |
| 71 | MARC0075425 | 60556 | 8 | 20831006 |
| 72 | MARC0093317 | 62211 | 8 | 76250536 |
| 73 | MARC0100227 | 62825 | 8 | 19268048 |
| 74 | DRGA0010843 | 39745 | 11 | 10278717 |
| 75 | H3GA0031835 | 44381 | 11 | 30363250 |
| 76 | INRA0036471 | 48917 | 11 | 38718827 |
| 77 | INRA0036473 | 48918 | 11 | 38737558 |
| 78 | ASGA0053943 | 26639 | 12 | 13873210 |
| 79 | ALGA0071850 | 10510 | 13 | 75371221 |
| 80 | CASI0009493 | 36044 | 13 | 24006591 |
| 81 | DBMA0000259 | 36247 | 13 | 75332168 |
| 82 | INRA0040988 | 49059 | 13 | 79284884 |
| 83 | ALGA0074932 | 10996 | 14 | 9016085 |
| 84 | ALGA0075064 | 11013 | 14 | 9800328 |
| 85 | ASGA0061144 | 27540 | 14 | 9672453 |
| 86 | ALGA0085842 | 12639 | 15 | 48564132 |
| 87 | ALGA0085893 | 12658 | 15 | 50398471 |
| 88 | ALGA0103648 | 15222 | 15 | 4300754 |
| 89 | ASGA0069860 | 28685 | 15 | 50306831 |
| 90 | H3GA0045081 | 45691 | 15 | 85329973 |
| 91 | ASGA0073467 | 29177 | 16 | 34320107 |
| 92 | ASGA0073470 | 29178 | 16 | 34391097 |
| 93 | MARC0093043 | 62184 | 16 | 35302397 |
| 94 | ASGA0077608 | 29613 | 17 | 46973188 |
| 95 | ALGA0099822 | 14668 | X | 43130553 |
| 96 | ALGA0099836 | 14676 | X | 48175934 |

Supplementary Table 2. The posterior probability any individual with log(LR) > 2 originates from the claimed breed.

|  | Contrasted breed | | | | | | | | | | | | | |
| --- | --- | --- | --- | --- | --- | --- | --- | --- | --- | --- | --- | --- | --- | --- |
| Claimed breed | BK | BS | DU | GLOS | HA | LR | LB | LW | MA | MS | MW | PI | TA | W |
| Berkshire | - | 0.999976 | 1 | 1 | 1 | 1 | 1 | 1 | 1 | 1 | 1 | 1 | 1 | 1 |
| British Saddleback | 1 | - | 1 | 1 | 1 | 1 | 0.999910 | 0.999811 | 0.999965 | 1 | 1 | 1 | 1 | 1 |
| Duroc | 1 | 1 | - | 1 | 1 | 1 | 1 | 1 | 1 | 1 | 1 | 1 | 1 | 1 |
| Gloucester Old Spot | 1 | 1 | 1 | - | 1 | 1 | 1 | 1 | 1 | 1 | 1 | 1 | 1 | 1 |
| Hampshire | 1 | 1 | 1 | 1 | - | 1 | 1 | 1 | 1 | 1 | 1 | 1 | 1 | 1 |
| Landrace | 1 | 0.993502 | 0.999727 | 1 | 1 | - | 1 | 0.999662 | 1 | 1 | 0.999997 | 1 | 1 | 0.991931 |
| Large Black | 0.999985 | 0.993539 | 0.999981 | 1 | 1 | 1 | - | 1 | 0.999929 | 1 | 1 | 1 | 1 | 1 |
| Large White | 1 | 0.994595 | 0.999749 | 1 | 1 | 0.984738 | 1 | - | 1 | 1 | 0.998120 | 1 | 1 | 0.999923 |
| Mangalica | 1 | 0.999995 | 1 | 1 | 1 | 1 | 1 | 1 | - | 1 | 1 | 1 | 1 | 1 |
| Meishan | 1 | 1 | 1 | 1 | 1 | 1 | 1 | 1 | 1 | - | 1 | 1 | 1 | 1 |
| Middle White | 1 | 0.999824 | 1 | 1 | 1 | 0.999913 | 1 | 0.999814 | 1 | 1 | - | 1 | 1 | 1 |
| Pietrain | 1 | 0.999813 | 0.999977 | 1 | 1 | 0.999549 | 1 | 0.999912 | 1 | 1 | 1 | - | 1 | 0.999922 |
| Tamworth | 0.999998 | 1 | 0.999987 | 1 | 1 | 1 | 1 | 1 | 1 | 1 | 1 | 1 | - | 1 |
| Welsh | 1 | 0.994867 | 0.998787 | 1 | 1 | 0.945844 | 1 | 1 | 1 | 1 | 1 | 1 | 1 | - |


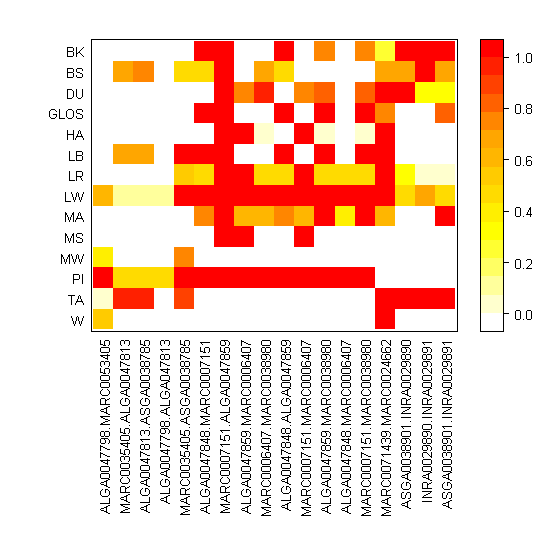


Supplementary Figure 1 Level of linkage disequilibrium (LD), measured using *r*^2^, between the 25 markers on chromosome 8 for each pig breed. *r*^2^ represents the correlation of allele frequencies between two loci such that SNPs in complete LD have a value of 1. The darker the colour, the higher the LD with white indicating no LD between a pair of SNPs.


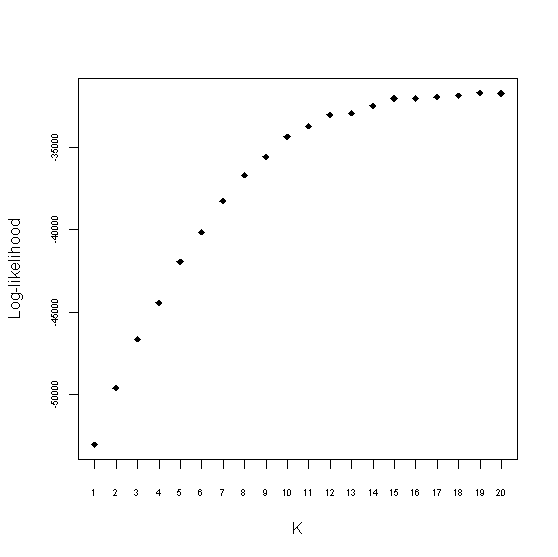


Supplementary Figure 2. Plot of the likelihood output of BAPS with increasing K value.
